# Female genital cutting (FGC) and the ethics of care: community engagement and cultural sensitivity at the interface of migration experiences

**DOI:** 10.1186/1472-698X-14-13

**Published:** 2014-04-24

**Authors:** Bilkis Vissandjée, Shereen Denetto, Paula Migliardi, Jodi Proctor

**Affiliations:** 1 Faculty of Nursing, Université de Montréal, PO Box 6128, Station Centre-Ville-Montréal, QC H3C 3J7, Canada; 2 SHERPA Research Centre and The Research Institute of Public Health at the Université de Montréal, Montréal, Canada; 3 Immigrant and Refugee Community Organization of Manitoba (IRCOM), 95 Ellen Street, Winnipeg, Manitoba R3A 1S8, Canada; 4 Sexuality Education Resource Centre (SERC) Manitoba, 200- 226 Osborne Street, North Winnipeg, Manitoba R3C 1V4, Canada; 5 School of Social Work, McGill University, 845 Sherbrooke Street West, Montreal QC H3A 0G4, Canada

**Keywords:** Female genital cutting, Female genital mutilation, Traditional practices, Migration, Public health, Ethics, Harm reduction, Community engagement, Cultural sensitivity

## Abstract

**Background:**

Female Genital Cutting (FGC) anchored in a complex socio-cultural context becomes significant at the interface of access of health and social services in host countries. The practice of FGC at times, understood as a form of gender-based violence, may result in unjustifiable consequences among girls and women; yet, these practices are culturally engrained traditions with complex meanings calling for ethically and culturally sensitive health and social service provision. Intents and meanings of FGC practice need to be well understood before before any policies that criminalize and condemn are derived and implemented.

FGC is addressed as a global public health issue with complex legal and ethical dimensions which impacts ability to access services, far beyond gender sensitivity. The ethics of terminology are addressed, building on the sustained controversial debate in regards to the delicate issue of conceptualization. An overview of international policies is provided, identifying the current trend of condemnation of FGC practices. Socio-cultural and ethical challenges are discussed in light of selected findings from a community-based research project. The illustrative examples provided focus on Western countries, with a specific emphasis on Canada.

**Discussion:**

The examples provided converge with the literature confirming the utmost necessity to engage with the FGC practicing communities allowing for ethically sensitive strategies, reduction of harm in relation to systems of care, and prevention of the risk of systematic gendered stigmatization. A culturally competent, gender and ethically sensitive approach is argued for to ensure the provision of quality ethical care for migrant families in host countries. We argue that socio-cultural determinants such as ethnicity, migration, sex and gender need to be accounted for as integral to the social construction of FGC.

**Summary:**

Working partnerships between the public health sector and community based organisations with a true involvement of women and men from practicing communities will allow for more sensitive and congruent clinical guidelines. In order to honour the fundamental principles and values of medical ethics, such as compassion, beneficence, non-malfeasance, respect, and justice and accountability, socio-cultural interactions at the interface of health and migration will continue to require proper attention. It entails a commitment to recognise the intrinsic value and dignity of girls’ and women’s context.

## 

 ‘*… Once the girl is cut, she is cut off …*’

Peter Kamuron, Human Rights Activist

## Background

In recent years, increased international migration from a wide variety of countries has exposed social and public health care professionals in host countries such as Canada to a diversity of issues associated with traditional practices adopted by selected migrant families as they progressively integrate into a new society. When it comes to such traditional practices, providing quality, ethical and safe health care highlights the responsibilities at different levels of the health care system of the host society. The objective of this paper is to examine the ethical and legal complexities raised by traditional practices such as female genital cutting (FGC) in multicultural health and social services practice settings. We argue that socio-cultural determinants such as ethnicity, migration, sex and gender need to be accounted for as integral to the social construction of FGC. The practice of FGC is often understood as a form of gender-based violence that often results in unjustifiable consequences among girls and women; yet, these practices are culturally engrained traditions with complex socio-cultural meanings calling for ethically and culturally sensitive health and social service provision. These meanings are often strongly juxtaposed alongside positive and less positive demonstrating that the intent/meaning of FGC in cultures needs to be well understood before any criminalization and condemnation policies are implemented.

The socio-cultural context must be addressed to ensure the reduction of harm in relation to systems of care, particularly to prevent gendered stigmatization of affected individuals. Social and health care professionals will need to strengthen their practice to reach the right balance in regards to their legal obligations along with their fundamental responsibility to provide equitable and compassionate care to all. Further to a general background on FGC practices, the cultural significance of FGC for practising communities will be illustrated with a case study discussion from the Sexuality Education Resource Centre Manitoba (SERC); these arguments will ground the analysis of the ethical complexity of FGC care and health service.

Ritual alteration of the genitalia of female infants, children, adolescents and adults has been a traditional practice in numerous cultures since antiquity. These practices have been documented in at least 26 countries in and around Sub-Saharan Africa. Practiced since the time of the pharaohs, FGC has been documented in a diversity of community groups from numerous religions, including but not limited to Animists, Catholics, Jews, Muslims, Protestants, and those without religious beliefs [1-3]. Having stated that, it is important to note that, contrary to popular belief, the primary motivations for these practices are often more anchored in cultural values than dictated by religious precepts.

The type of FGC procedures varies not only across countries, but also within countries, across ethnic groups and within cultural communities [4]. The World Health Organization (WHO) classifies the alteration of the genitalia of female infants, children, adolescents and adults into four types: type one refers to the partial or total removal of the clitoris and, in very rare cases, only the prepuce; type two refers to the partial or total removal of the clitoris and the labia *minora*, with or without excision of the labia *majora*; type three refers to the stitching/narrowing of the vaginal opening through the creation of a covering seal; and finally, type four refers to all other procedures used to alter the female genitalia for non-medical purposes, e.g., pricking, piercing, incising, scraping and cauterizing the genital area [5].

Within many patriarchal societies, the tradition of FGC is intended to ensure control of female sexuality, chastity and the honour of the community. However, FGC practices hold many additional cultural meanings, including but not limited to the preservation of group identity; a rite of passage ensuring social transition from one status level to another; preservation of virginity and family honour; and the furthering of marriage goals, including the enhancement of sexual pleasure for men. Though cultural meanings associated with the practice are diverse, it is clear that FGC practices are often viewed as a social good, essential in the socialisation of girls [1,2,6,7].

Given the evidence of physical harm caused by FGC, selected reactions from practicing communities point to the potential of social exclusion and marginalization of women (among other socio-cultural consequences) that may result when girls or women are not excised or infibulated, potentially bringing greater harm [7-10]. Though the medico-physical consequences of FGC are increasingly documented in international literature, the complex nature of the socio-cultural effects associated with these practices or lack thereof require further discussion, especially when anchored within the intricate trajectory of integration into a new society.

### The ethics of terminology

The language used to describe these practices remains controversial and requires careful ethical consideration. The term ‘Female Genital Mutilation,’ formerly adopted by the United Nations (UN) calls attention to the gravity of the harm caused by FGC practices. ‘Female Genital Mutilation (FGM)’ is the terminology used within campaigns to end these practices by anti-FGC advocates from practicing countries of origin and the western world. FGM terminology positions the practice of FGC as an extreme human rights violation. This term is often perceived as inflammatory, judgemental and stigmatising, particularly for women previously exposed to the practice who do not view their bodies, or the bodies of their daughters, as mutilated [3]. The implication within this terminology is that FGC is practiced as an act of intentional violence against female children, adolescents and women. Those who do not understand FGC as such an act, but as a valued cultural tradition, may experience the language of “mutilation” as alienating [7,9-11]. The delicate challenge of reconciling respect for cultural values associated with these practices and addressing their perceived harmful effects on health is evident in this discrepancy between the intent and impact of language.

‘Traditional women’s practices,’ ‘Traditional health practices,’ and ‘Initiation,’ are some of the preferred terms identified by individuals who subscribe to the socio-cultural benefits from these practices. Chalmers & Omer Hashi [10] as well as Vissandjée et al. [7] conducted focus groups with overall 600 women from different practising countries living in Canada which revealed “circumcision” to often be the preferred terminology. Several other authors have also identified “circumcision” as an alternative term, yet this term has been argued to trivialise the procedure, falsely attributing to FGC the legitimacy afforded to male circumcision within the West [12,13]. “Female Genital Cutting (FGC)” and “excision and infibulation” have been identified as more neutral, ethically sensitive terminology [4,6]. For the purpose of this chapter, we will use FGC as a term comprising procedures which alter the female genital organs for cultural or non-therapeutic reasons.

## Discussion

### Criminalization and condemnation: an overview of international policies

In multi-ethnic societies across the western world, professionals in the field of health and social services are faced with an increasing number of women, men, and families originating from countries where practices such as FGC are common [14-18]. International trends of migration contribute to the growing controversy regarding traditional practices as they meet up with host society’s cross-cultural imperatives in the health care system. As such, health care professionals are required to deal with the ethical complexity of navigating through their own personal identity and culture, most often in opposition with the identity and cultural processes of the women and men migrants they are meant to serve while bound by their legal and professional guidelines [19].

### Criminalization and condemnation: an overview of Canadian policies

In 1997, the Canadian Criminal Code was amended through bill C-27 to declare acts of FGC a criminal offence. Those caught performing these acts are subject to prosecution. If health and social service providers believe a female child is at risk of the practice, or has already undergone the practice, they are required to report to relevant statutory bodies under provincial child welfare legislation [4,20]. Canada’s legal position is consistent with the WHO, World Medical Association and the International Federation of Gynaecology and Obstetrics and Society of Obstetricians and Gynaecologists of Canada which have all condemned the practice. FGC practices are becoming increasingly prohibited by law, both in countries where it is traditionally practised and in countries of immigration [8,15,18].

Legal condemnation has had notable socio-cultural consequences in host countries such as Canada. Macklin documented the experience of a Sudanese family in St. Catharine’s, Ontario who were charged with having the procedure performed on their daughter in Canada, by an unidentified practitioner at the age of 11 [20]. Though the charges were eventually withdrawn by the prosecution due to lack of evidence, the state intervention in this case resulted in the detention of the parents in custody and the apprehension of their two children by child welfare authorities. Macklin notes that police, child welfare authorities, and lawyers acted with cultural insensitivity. It is additionally highlighted that long term emotional and social damage to the complainant, the accused and the family was incalculable.

Under the Canadian Medical Association’s (CMA) Code of Ethics section ‘Responsibilities to Society,’ article 41 states that health professionals “must recognise that community, society and the environment are important actors in the health of individual patients” [19], p. 3. In instances where health care professionals are faced with the legal obligation to notify statutory bodies of the occurrence or risk within the host society of FGC, ethical considerations of article 41 need to be carefully weighed with their legal requirement to report. Furthermore, the CMA Code of Ethics section ‘General Responsibilities’ article 14 states that health professionals must “take all reasonable steps to prevent harm to patients” [19], p. 2. This is where the relative nature of the definition attributed to harm becomes of concern. Selected communities do not necessarily view the practice of FGC (more often, re-infibulation post-delivery) as potentially harmful; rather the lack thereof may lead to psychosocial harm experienced by girls and women at risk of being ostracised by members of their community.

Indeed, many authors converge by highlighting the fact that criminalising practices of FGC may have unintended adverse effects, such as the risk of clandestine practice of FGC within host countries, alternate practices such as a ritual nick or that parents send their girl child to the home country to undergo these practices in unsafe conditions [6,9,15].

Issues associated with the practices of FGC in the West have notable gray areas, including but not limited to the requests for re-infibulation of the vaginal canal after childbirth or notification of the courts about previously performed practices. Re-infibulation within medical settings has been a common occurrence across the western world, often brought forward as a potential harm-reduction strategy in order to minimise health hazards associated with potentially risky behaviours. Such practice is argued to offer safer and culturally acceptable alternatives that bear the least amount of psychosocial harm [17]. Such clinical requests call for an increased awareness of health care professionals’ ethics and responsibilities to give the best care possible while being sensitive to one’s life context; such clinical requests may be considered by many to be contrary to professional medical ethics, yet the extent to which re-infibulation constitutes a breach of law remains unclear in many Western countries. It should be noted that legislation condemning FGC may not resolve the ethical difficulty faced by health care professionals even if increasingly anchored as a human rights abuse with the responsibility to “refuse to participate in or support practices that violate human rights” [19], p. 1.

Having stated that, a recent policy statement and guidelines from the SOGC clearly states that requests of re-infibulation must be denied [4]; on the other hand, one needs to remember to trust a medical judgment call whether or not to perform re-infibulation, beyond the ethical and legal controversy surrounding the practice if it is assessed to be for the ‘patient’s good’ and that ‘harm’ will be prevented [11,14].

### Criminalization and condemnation: an international controversy

In Italy, the United States and the Netherlands, proposed policy developed in partnership with selected practising communities has provoked great controversy. In an historic case in Seattle in 1996, physicians at the Harborview Medical Centre suggested a symbolic procedure of pricking the clitoral hood be performed in order to appease traditional Somali families. It was argued that such a procedure would be less invasive than circumcision (as performed on male babies) and would minimise the risk of individuals seeking FGC services illegally within the host country or sending the child abroad to the home country. Though this proposal was approved by a committee of health professionals and medical ethicists it was met with public outcry which prevented it from moving forward.

Equality Now (US based) highlighted that, in 2010, further to their policy statement in reference to paediatricians’ ‘nicking’ of girls genitalia, ‘encouraging paediatricians to perform this practice with the umbrella of ‘cultural sensitivity’ is simply a shocking lack of understanding of girls’ fundamental right to bodily integrity and equality. Equality Now suggested to members of the American Academy of Paediatricians, an awareness-raising discussion in collaboration with practising immigrant communities about these highly sensitive and ethical practices leading to potentially harmful consequences including tampering with a recognised human rights violation against girls and women [21,22].

A similar proposal was put forward in Florence, Italy in 2003 by the Reference Centre for Preventing and Curing FGM of the Department of Gynaecology, Perinatology and Reproduction Physiology. The suggested alternative ritual was a prick of the clitoral hood with a small needle, done under local anaesthesia on children old enough to provide consent. An Italian bioethics committee judged this proposal to be ethical, yet international public protest prevented its adoption [1].

Selected analyses of a human rights framework juxtapose western acceptance of male circumcision and female genital constructive surgery in European and North American countries with clear condemnation of FGC practices among immigrants from practising countries [23]. Very recently, a German court in Cologne ruled against circumcising young boys for religious reasons [24,25]. The Court found that the ‘child’s fundamental right to bodily integrity’ was more important than his parents’ fundamental rights to religious freedom. It should be noted that Germany does not carry a law against male circumcision, as opposed to the one against FGC, creating additional uncertainties about procedures related to both sexes. After months of debate, in December 2012, German lawmakers overturned this law granting parents the right to authorize male circumcision by a trained practitioner. Similarly, in the US, non-therapeutic circumcision of male children is part of basic health care; in regards to FGC, a number of US states have adopted laws against FGC [22,26]. In the same vein as the arguments made throughout this paper, Equality Now reinforces the fact that it is critically important for relevant local and community groups to be involved at all levels to address this sensitive issue within the diversity of relevant communities. Culturally sensitive awareness-raising, education and outreach programs need to be sternghtened in order to protect a new generation of american girls. To be effective, approaches addressing FGC need to be holistic and include education and outreach components as well as measures for legal protection and accountability [22].

While getting better informed and trained about the consequences of these practices and the premises underlying them, health care professionals need to reflect upon the intricate ethical complexity of the contribution of determinants such as ethnicity, migration, sex and gender in the social construction of FGC practices among migrants in western countries where there is, in pllel, a rise in female genital reconstruction surgeries. Just as Johnsdotter & Essén [27] stated: “… *procedures involving genital modifications are intertwined with political considerations. They are never purely about anatomy and physiology but are intrinsically entangled with cultural norms, identity and ideology. The pricking of the clitoral hood among women from countries of Africa is condemned, while reduction of clitoral tissue among women across countries of Europe is legal and accepted …*” [27], p. 35.

In 2011 in Indonesia, though FGC was banned in 2006, guidelines to physicians on how to perform FGC were issued by the Indonesian Ministry of Health. Public statements of strong concern followed this release by a number of medical experts and rights groups who argued that release of the guidelines was responsible for an increase of FGC practice in medical settings. Of concern was also the issue that guidelines could well be misinterpreted as an endorsement of the procedure, combined with an enticement for doctors to encourage the practice [28].

### At the interface of care: culturally and ethically sensitive quality of care for all

The fear of stigmatisation within the host society cultural norms in regards to the integrity and rights of girls’ and women’s bodies as well as the overt perceptions that FGC is a deviant practice that requires criminalisation has been documented as inadvertently limit women and men from accessing needed quality health services [7-9]. In addition, intercultural communication difficulties stemming from linguistic and other cultural barriers have been identified as key deterrents for many immigrant women and men in accessing health services [29-32]. Establishing proper and quality communication as the basis of an ethical clinical situation has been highlighted in ample empirical evidence as well as selected codes of ethics for health care professionals such as nurses and physicians in the context of linguistic barriers [33].

A number of women emigrating from practising countries may have already undergone FGC upon arrival [34]. At some point, many of these women will be required to use the social and health care system, particularly during pregnancy and childbirth. While studies have demonstrated the immediate harmful health effects of FGC practices, in terms of uncontrolled bleeding and infection, selected long-term effects are not well understood and may vary. Some of the outcomes include vaginal infections, difficult second stage delivery, as well as ill-sustained menstrual and sexual pain. Ill-health outcomes may affect not only those new to Canada but also those who have been in Canada for a long time but are slowly getting to know the health care system due to lack of knowledge in regards to access and general discomfort with health care providers [7,35].

It has also been documented that women having experienced these traditional practices tend to not avail themselves of other services such as sexual health clinics or pain management, not recognising that the pain may not be a “normal” condition, whether it be provoked during sexual activities or a chronic sensation. When Einstein [36] asked women about pain during any of their daily experiences, they considered them negligible - just what is ‘normal’ and what every woman has. However, when women were actually tested for pain in the vulvar region, Einstein found that all of her participants had at least one area of the vulva in which the pressure-pain threshold was lower than that for Canadian women with vulvar vestibulitis, a chronic vulvar pain condition. Einstein concludes by suggesting that her participants had chronic pain but to them it was just part of normal life [36].

European and North American studies of health care professionals’ knowledge, perception and management of birth for women who have experienced FGC have found significant gaps in knowledge and clinical practice related to the delicate and complex nature of care required [17,33,37,38]. Unfamiliarity, subtle discomfort and lack of guidelines or uptake of the latter may induce serious mismanagement of infibulation during delivery associated with the risk of psychological harm [37]. In addition, unnecessary caesarean sections have been reported among women from practicing countries due to the lack of familiarity and overall discomfort of Canadian health care professionals with the practice of infibulation [7,10]. Similar findings have been noted in Germany and other Western European countries [11]. Chalmers & Omer Hashi [10] have reported that 87.5% of the 432 Somali women interviewed in Ontario birth experiences in Canada have disclosed unpleasant and hurtful comments made by caregivers during delivery. The women interviewed reported verbal expressions of negative shock and a sense of disgust by selected caregivers, perceived to a certain extent as a lack of respect and privacy, especially when in some instances, colleagues are called upon to ‘take a look’ without prior request or permission [10].

Under the section ‘Initiating and Dissolving a Patient-Physician Relationship’ of the CMA Code of Ethics [19], article 17 states that health professionals are ethically bound not to discriminate: ‘… *in providing medical service, do not discriminate against any patient on such grounds as age, gender, marital status, medical condition, national or ethnic origin, physical or mental disability, political affiliation, race, religion, sexual orientation, or socioeconomic status…*’ However subtle, the sense of perceived disrespect towards a girl or a woman on grounds of her having experienced FGC, is a violation of article 17.

In order to softly navigate through delicate subjects such as FGC at the interface of sex, gender and migration experiences while seeking care in host countries such as Canada, it is necessary to strengthen competence, congruency and compassion, along with the uptake of sensitive guidelines in partnership between medical and community organisations. The Sexuality Education Resource Centre (SERC), based in Winnipeg Manitoba, is among selected organisations in Canada that aims to provide sensitive and culturally competent care to girls, women and families when needed. A case study illustrating the interactions between SERC and women they attend to will allow the reader to anchor the discussion provided above as SERC strives to give voice to those living experiences associated with FGC practices. The aim of this section is to enhance the importance of a deeply sensitive and reflexive practice with an illustration of the complexity of socio-cultural issues raised via a dialogic process between a diversity of women and men and members of a community organization.

### Our Selves, Our Daughters: an illustration

Despite the fact that immigration is a notable reality in places like Toronto, Vancouver and Montreal, Winnipeg is increasingly receiving its fair share of immigrants. In Manitoba, immigration has increased exponentially in the past decade due to highly successful provincial programs and policies, most notably the Provincial Nominee Program along with the long standing commitment to assist in the relocation of refugees in Canada. Over the years, a number of families from select countries in Africa have sought refuge in Manitoba [39]. In the past decade, at least seven out of the top ten source countries of refugees have been African countries, namely Ethiopia, Sudan, Eritrea, Somalia, Egypt and Sierra Leone, where prevalence levels of FGC range from 74.3% to 97.9% [40].

SERC with centres in Winnipeg and Brandon has been attending to the needs of immigrants and refugees, primarily newcomers, for 25 years. SERC's main mission is to promote sexual health through education. Central to SERC’s work in education and prevention is an analysis of sexuality in its broadest sense that takes into account the intersections of sexuality and culture, values, gender, identity and migration. Since 2009, SERC has been working closely with specific migrant communities in Winnipeg to tackle this complex, rich and deeply culturally entrenched tradition. SERC seeks to work closely with women and their families, community members as well as service providers in order to reduce any risks associated with the practice of FGC as the families integrate into a new society with its specific cultural and at times contrasting values in regards to FGC. Addressing such a controversial issue has required a process-oriented, iterative approach to build community trust and successful partnerships, prerequisites for successful engagement on this sensitive and potentially stigmatizing issue.

Additional context is provided by the fact that SERC subscribes to harm reduction, health promotion and illness prevention when it comes to sexual and reproductive issues. The following definition of harm reduction has been adopted by SERC as it applies to a range of sexual and reproductive health issues including FGC: a set of strategies and tactics that encourages people to reduce harm to themselves and their communities, through the sharing of relevant information, facts and practical material tools that will allow them to make informed and educated decisions. It recognises the competency of their efforts to protect themselves, their loved ones and their communities [41].

Applying these approaches to this community-based project meant first learning more about the practice through a community lens and through that process engaging community. To this end, the “Our Selves, Our Daughters” project began with a community-based research process. Community members were hired to conduct different phases of the research. Community views were elicited through gender-segregated focus groups (7 with 48 women, 1 with 9 young men, 1 with 7 young women, 2 with 19 men) and individual interviews with eight community members in leadership positions (5 men and 3 women), and professed religious leaders (6 men and 1 woman). Core members of the team conducted thematic analysis of the data, which were presented to participants invited to a feedback session. Further analysis was conducted with their feedback to refine our themes.

Addressing this taboo and culturally sensitive issue has enabled SERC to uncover assumptions and help readjust theworld-views of those coming from non-FGC practicing communities, steeped in Western feminist thought. While delivering service provider training, SERC facilitators tended to wrongly assume that women affected by FGC were knowledgeable of harms associated with the practice. Discourses are discordant.

“-It can cause problems to the health of the woman. The sexual desire also can be reduced. Even it can also affect the fertility. She can become infertile.

- I didn’t get your point. Do you mean if some organs are injured as a consequence of female circumcision?

- No, I think she can’t become infertile because of circumcision. The fertility part of the woman’s body is not on the place where circumcision is done.

- She may experience problems during labour.

-Maybe.

- I don’t think it brings problems to labour. Labour is a natural process. So this labour problem happens naturally. There is no link between circumcision and labour. It might have other problems.

- [Circumcision] is normal in [my country], Does it have side effects?

- I remember there was something they say ‘fistula,’ fistula for urine and fistula for stool. That is because of circumcision.

- What is fistula?” [42], p. 21.

Converging with previous studies, a large number of women did not realize that the long-term health impacts that some were experiencing may have been linked to FGC, such as recurrent UTIs, painful menstruation/blood retention, constantly sensitive perineum, and complications during childbirth. It was accepted that if a woman experienced pain and illness, they were as likely to be caused by a curse or evil spirits. Many women attending SERC’s workshops shared their belief in the role of supernatural forces regulating cause and effect in many aspects of their lives.

Given that many participants were misinformed about the practices themselves, about rights and access to quality health care in Canada as well as about interpretation services, SERC facilitators built specific training material for women and men including the function and configuration of women’s internal organs, maturation of the body through menstruation, conception, pregnancy, childbirth and menopause. Women's reactions were varied, upon receipt of this new information. The strongest reaction was illustrated by women expressing regrets, stating that if they had known then what they were learning now, they would have made different decisions about their bodies. Other women were exposed many times to anti - FGC campaigns and to changing laws in their country of origin or during migration; they knew; but, they could not act much in this regard. For other women, FGC practices were not viewed as harmful practices; they were perceived to be simply a necessary step to ensure personal, social, legal and economic standing for a woman in her community and society at large:

-“A mother was doing FGC because she loves her child and wants to protect her child. We are not ashamed of our culture, we are proud” [43], p. 27.

-“Acknowledge that she loves her children. Don’t victimise women” [42], p. 28.

-“Here the culture is different from back home. At home – if a child is not circumcised there is stigma. There is less chance that she will get married. When men sees she is not circumcised she will have an un-peaceful life” [43], p. 28.

-“The reason for female circumcision is to make her polite, to prevent her from becoming hyper, to prevent her from looking [for] extramarital sex, to prevent her from misbehaving” [42], p. 11.

-“I know they were doing it to protect their daughters; they had a strong belief that their daughter won’t be raped easily” [42], p. 29.

Many women and men shared these viewpoints. As one religious leader shared on the issue of change:

-“We have to weigh…Who we listen to? The oral culture that has been passed down from our elders for thousands of years … or this more recent information?” [44], p. 28.

Understanding ‘harm’ related to FGC may seem self-evident to a Western audience. In most FGC practicing communities, ‘harm’ is deeply anchored in cultural norms described by many women, particularly elders, that a woman's life is inextricably linked to suffering. In societies where childbirth and practices such as FGC occur without anaesthetics, enduring pain and suffering is expected. Women were described as the “root” or “heart” of the family and community by male participants in group discussions at SERC. As such, women are expected to put the needs of others before their own in addition to downplaying pain:

-“Even if we are hurt, as a woman we don’t open our mouth and say it” [42], p. 12.

-“I heard many divorce are resulted because of sexual issues. In our country if you ask a woman what was the cause of the divorce she says [word in own language], ‘…I don’t want to keep living there…’She doesn’t open her mouth and say. When we don’t talk, the trauma may not be expressed” [42], p. 12.

Such perceptions represent a fair challenge for organisations like SERC to create and maintain a space in which women can safely disclose experiences both positive and adverse without breaching this valued silence seen by some cultures as intrinsic to a women’s identity.

## Summary

### Ethically sensitive approach: reframing the concept of ‘harm’

Few topics elicit such a strong, visceral reaction among women and men from non-practicing countries, as do FGC practices. The fact that many women are the keepers of this tradition, that the practice is so widespread among some groups, given the sub-optimal conditions under which the practice occurs, and the fact that it mostly affects girls creates challenges for health care and community-based providers to give ethically, gender sensitive, non-judgmental care; this is key to the success of SERC’s community-based work as change is progressively and carefully promoted. The issues raised above are key ‘ingredients’ in the training sessions that SERC is engaged in with a diversity of providers along with women themselves, while core information about FGC, diverse cultural meanings and social constructions are shared.

In order to be non-judgmental and ethical, to avoid further marginalization of newcomer women, SERC’s providers had to reflect upon their own feelings and reactions as well as beliefs and values, recognise them and suspend judgement. The presence of an in-community facilitator was essential in these teachings, as she modelled how to reflect the normalcy of FGC in practicing cultures. In addition to this most valued presence, the briefings that were held allowed providers to move away from error-inducing dichotomies such as ‘freedom’ vs. ‘oppression’, and ‘them’ vs. ‘us’ as well as ‘sensitive’ vs. ‘insensitive’ towards quality of service and care for everyone within an environment that would be as bias free as possible [45]. As providers become progressively aware of the continuum of beliefs and thoughts about the traditional practices, group discussions about a possible shift in these thoughts are held as information is shared with a diversity of women and men.

Similarly, a partnership in Switzerland, between community providers, professional actresses and amateur actors with a migration background have devised a play in English, French and Somali to increase public awareness, without accusing or judging, thereby building trust for the rounds of discussions that follow the performance [46].

In its mission to establish trust and freedom of conduct while sharing information and training women and providers, one of the challenges SERC faced was to carefully manage potential backlashes within and between members of community groups. SERC has begun to integrate issues associated with sexuality in general while increasing the time allocated to the training in order to allow for sharing and building constructively on potential emotional reactions. As discussed above, the involvement of statutory agencies such as child protection services and the police during selected training sessions have been perceived as an added value anchored in the principles of ethical collaborative strategies. Several initiatives are underway in Winnipeg to build a proper understanding of the role these agencies play in prevention, protection and support as newcomers are simultaneously encouraged to express their views about these institutions. Of interest, no such institutions have yet participated in SERCs’ group discussions so as to not affect the trusting relationships.

Working partnerships between the public health sector and community based organisations with a true involvement of women and men from practicing communities will allow for more sensitive and congruent clinical guidelines. In order to honour the fundamental principles and values of Canadian medical ethics, such as compassion, beneficence, non-malfeasance, respect, and justice and accountability, the complex nature of socio-cultural interactions at the interface of health and migration will continue to require proper attention.

One of the most telling prescriptions from the Canadian Medical Association (CMA) Code of Ethics, article 12, states: “to practice the profession of medicine in a manner that treats the patient with dignity and as a person worthy of respect” [19], p. 1. It entails a commitment to recognise the intrinsic value and dignity of women’s context. It has been argued earlier that selected, at times unintended, reactions from health care professionals may lead to perceptions of stigmatization by some women from practicing communities.

As a final note, it is therefore not superfluous to reiterate the importance of the obligation of health care professionals to provide compassionate and ethical care, especially when clinical situations are complex involving personal, community and legal consequences of a single clinical decision.

‘ … *It is unacceptable that the international community remains passive in the name of a distorted vision of multiculturalism. Human behaviour and cultural values, however senseless or destructive they may appear from the personal and cultural standpoints of others, have meaning and fulfil a function for those who practice them. However, culture is not static but is in constant flux, adapting and reforming. People will change their behaviour when they understand the hazards and indignity of harmful practices and when they realize that it is possible to give up harmful practices without giving up meaningful aspects of their culture*…’

Joint Statement (August 2007)

UNFPA, UNICEF and WHO at the Global Technical Consultation in Addis Ababa, Ethiopia

‘ … *Culture is a matrix of infinite possibilities and choices. From within the same culture matrix we can extract arguments and strategies for the degradation and ennoblement of our species, for its enslavement or liberation, for the suppression of its productive potential or its enhancement…*’

Wole Sovinka, Nigerian Nobel Laureate

## Abbreviations

CMA: Canadian Medical Association; FGC: Female genital cutting; FGM: Female genital mutilation; SERC: Sexual Education Resource Centre Manitoba; UNICEF: The United Nations Children’s Fund; SOGC: Society of Obstetricians and Gynaecologists of Canada; UNFPA: The United Nations Population Fund (*UNFPA*), formerly the United Nations Fund for Population Activities; WHO: World Health Organization.

## Competing interests

The authors declare that they have no competing interests.

## Authors’ contributions

The four authors participated equally in the development of the outline for this paper. BV and JP (under BV’s supervision) held primary responsibility in the gathering of all relevant literature in the actual writing of the ‘background’ and ‘discussion’ sections of the paper; SD and PM held primary responsibility for carrying out and describing the case study, including gathering and inclusion of relevant literature. All authors read and approved the final manuscript.

## Pre-publication history

The pre-publication history for this paper can be accessed here:

http://www.biomedcentral.com/1472-698X/14/13/prepub
